# Risk factors and prognosis of young stroke. The FUTURE study: A prospective cohort study. Study rationale and protocol

**DOI:** 10.1186/1471-2377-11-109

**Published:** 2011-09-20

**Authors:** Loes CA Rutten-Jacobs, Noortje AM Maaijwee, Renate M Arntz, Mayte E Van Alebeek, Pauline Schaapsmeerders, Henny C Schoonderwaldt, Lucille DA Dorresteijn, Sebastiaan Overeem, Gea Drost, Mirian C Janssen, Waander L van Heerde, Roy PC Kessels, Marcel P Zwiers, David G Norris, Maureen J van der Vlugt, Ewoud J van Dijk, Frank-Erik de Leeuw

**Affiliations:** 1 Donders Institute for Brain, Cognition and Behaviour, Centre for Neuroscience, Department of Neurology, Radboud University Nijmegen Medical Centre, PO Box 9101, 6500 HB Nijmegen, The Netherlands; 2 Radboud University Nijmegen Medical Centre, Department of Medical Psychology, Radboud, 6500 HB Nijmegen, The Netherlands; 3 Medisch Spectrum Twente, Department of Neurology, P.O. Box 50 000, 7500 KA, Enschede, The Netherlands; 4 Radboud University Nijmegen Medical Centre, Department of Internal Medicine, PO Box 9101, 6500 HB Nijmegen, The Netherlands; 5 Radboud University Nijmegen Medical Centre, Central Laboratory for Haematology, PO Box 9101, 6500 HB Nijmegen, The Netherlands; 6 Radboud University Nijmegen Medical Centre, Department of Geriatrics, Radboud, 6500 HB Nijmegen, The Netherlands; 7 Donders Institute for Brain, Cognition and Behaviour, Centre for Cognition, Radboud University Nijmegen, 6500 HE, The Netherlands; 8 Donders Institute for Brain, Cognition and Behaviour, Centre for Neuroscience, Department of Psychiatry, Radboud University Nijmegen Medical Centre, The Netherlands; 9 Donders Institute for Brain, Cognition and Behaviour, Centre for Cognitive Neuroimaging, Radboud University Nijmegen, 6500 HE Nijmegen, The Netherlands; 10 Radboud University Nijmegen Medical Centre, Department of Cardiology, PO Box 9101, 6500 HB Nijmegen, The Netherlands

## Abstract

**Background:**

Young stroke can have devastating consequences with respect to quality of life, the ability to work, plan or run a family, and participate in social life. Better insight into risk factors and the long-term prognosis is extremely important, especially in young stroke patients with a life expectancy of decades. To date, detailed information on risk factors and the long-term prognosis in young stroke patients, and more specific risk of mortality or recurrent vascular events, remains scarce.

**Methods/Design:**

The FUTURE study is a prospective cohort study on risk factors and prognosis of young ischemic and hemorrhagic stroke among 1006 patients, aged 18-50 years, included in our study database between 1-1-1980 and 1-11-2010. Follow-up visits at our research centre take place from the end of 2009 until the end of 2011. Control subjects will be recruited among the patients' spouses, relatives or social environment. Information on mortality and incident vascular events will be retrieved via structured questionnaires. In addition, participants are invited to the research centre to undergo an extensive sub study including MRI.

**Discussion:**

The FUTURE study has the potential to make an important contribution to increase the knowledge on risk factors and long-term prognosis in young stroke patients. Our study differs from previous studies by having a maximal follow-up of more than 30 years, including not only TIA and ischemic stroke but also hemorrhagic stroke, the addition of healthy controls and prospectively collect data during an extensive follow-up visit. Completion of the FUTURE study may provide better information for treating physicians and patients with respect to the prognosis of young stroke.

## Background

Up to 12% of all stroke occur in patients between 18-50 years ("young" stroke) [1], affecting about 5000 patients each year in the Netherlands and about 2 million young people each year worldwide. In a substantial proportion of roughly one third the etiology remains unelucidated. In terms of prognosis a "young" stroke has a dramatic influence on independency and quality of life as it occurs in the period of life that people start to form families, make decisive career moves, and have an active social life. Uncertainty about long term prognosis affects choices and planning affiliated with these life events.

Whereas risk factors and prognosis in patients who develop a stroke at higher ages (usually over 70 years) are among the best studied topics in clinical medicine, this does not hold true for young stroke. At higher ages, almost all risk factors have atherosclerosis in their final common pathway. However, this cannot simply be extrapolated to young stroke as the underlying cause of stroke is usually different from that in elderly and may therefore also have a different prognosis both with respect to functional stroke outcome as to risks of recurrent stroke or other major vascular events. Even more, the identification of risk factors for young stroke so far has often been based on the occurrence of presumed risk factors in consecutive series of young stroke patients, without methodological sound comparison with controls.

The "long-term" perspective in an on average over 70 years "old" stroke patient differs from that of a 30 years "young" stroke patient, and particularly studies with a long-term follow-up of more than 10 years are lacking in the young stroke field. Studies thus far, usually with a mean follow-up duration of less than 7 years, report highly variable post-stroke mortality and risk of incident vascular disease [2-7]. These large differences across studies are well explained because young stroke is a heterogeneous disease and most studies were small, had different selection criteria, did not investigate patients in person but relied on telephone interviews and outcome assessments and follow-up planning was not uniform and often suboptimal. Although stroke includes both ischemic and hemorrhagic stroke, almost all studies have excluded the investigation of etiology and prognosis of young hemorrhagic stroke.

Except for recurrent vascular disease and persistent motor and language impairments, post-"young" stroke quality of life will most likely also be determined by cognitive dysfunction, depressive symptoms, fatigue, and specific post-stroke complications such as epilepsy, because those determine the ability to (return to) work and to have a normal family and social life. Data on those aspects in the very long-term follow-up of young stroke patients are even more scarce.

Although the absolute number of young stroke is lower than stroke among the elderly, the total number of years that young stroke patients as a whole will live with the consequences of the stroke exceeds that of older stroke survivors due to far longer survival.

This justifies a properly designed and executed study on risk factors and prognosis of young stroke, compared with controls. We therefore set up the ***FUTURE ***study (***F****ollow**-U**p *of ***T***ransient ischemic attack and stroke patients and ***U***nelucidated ***R***isk factor ***E***valuation study), the largest single-centre prospective cohort study on risk factors and prognosis of young TIA, ischemic stroke and hemorrhagic stroke patients (n = 1006) and controls.

## Methods/Design

The FUTURE study is a prospective cohort study that aims to investigate the causes and consequences of a young stroke. The Medical Review Ethics Committee region Arnhem-Nijmegen approved the study.

### Patients

The department of neurology has a long-standing interest in the etiology and prognosis of young stroke and therefore maintains a prospective registry of all consecutive young stroke patients with a standardized collection of baseline and clinical characteristics (see baseline) since the 1970'ies [8]. For the current *FUTURE*-study, all consecutive TIA, ischemic stroke patients with presumed arterial origin or those with an intracerebral hemorrhage that sought medical attention for these disorders at the department of neurology of the Radboud University Nijmegen Medical Centre between 1-1-1980 and 1-11-2010 will be eligible for participation in the study.

#### Inclusion criteria

1. TIA, ischemic stroke of presumed arterial origin or intracerebral hemorrhage

2. Date of onset between 1-1-1980 and 1-11-2010

3. Age 18-50 at onset

#### Exclusion criteria

1. Traumatic hemorrhagic stroke

2. Intracerebral hemorrhage in known cerebral metastasis or primary brain tumor

3. Ischemic/hemorrhagic stroke due to cerebral venous sinus thrombosis

4. Intracerebral hemorrhage due to ruptured cerebral aneurysm

5. Any subarachnoid hemorrhage

6. Retinal infarct

TIA was defined as a rapidly evolving focal neurological deficit with no other than a vascular cause lasting less than 24 hours. Stroke was defined similarly, but with symptoms lasting more than 24 hours. On the basis of radiological findings, stroke was further subdivided into hemorrhagic and ischemic stroke.

As the diagnostic process may have changed during more than 30-year period all initial diagnoses were reviewed by a panel of two experts from a pool of four (FEdL, EvD, RA, LJD) and in cases of disagreement a consensus meeting was held to adjudicate the event.

1006 patients who had sought medical attention at our University Medical Center between 1-1-1980 and 1-11-2010 fulfilled inclusion and exclusion criteria for our study. Characteristics of our baseline population (at the time of their qualifying event) are reported in table 1.

**Table 1:** Baseline population characteristics

	Total population	Time of index event		
		1980-1989	1990-1999	2000-2010
n	1006	223	249	534
Male, n (%)	470 (46.7)	110 (49.3)	128 (51.4)	232 (43.4)
Age at index event, mean (sd)	40.2 (7.9)	39.3 (8.3)	39.7 (8.6)	40.8 (7.4)
Index event				
TIA, n (%)	277 (27.5)	52 (23.3)	40 (16.1)	185 (34.6)
Infarction, n (%)	630 (62.6)	146 (65.6)	189 (75.9)	295 (55.2)
Hemorrhage, n (%)	99 (9.8)	25 (11.2)	20 (8.0)	54 (10.1)

### Controls

Control subjects will be recruited among the patients' spouses, relatives or social environment.

They have to be at least 18 years old without a history of any TIA or stroke before the age of 50 at the moment of inclusion.

### Baseline

At baseline (during the occurrence of the qualifying event for the study) a minimal dataset has been collected that consists of demographics, stroke subtype, risk factors and additional investigations (table 2). The completeness of the baseline dataset varies among patients due to changes in standard diagnostic procedures over the last thirty years.

**Table 2:** Schedule of assessments in the FUTURE study

Assessment	Baseline	Follow-up
**Demographics**		
Ethnicity		X
Education		X
Marital status		X
Social/living status		X
**Stroke Characteristics**		
Qualifying event	X	
Symptoms at onset	X	
Discharge date and destination	X	
TOAST	X	
NIHSS at admission and at discharge	X	
Modified Ranking Scale at discharge	X	
**Medical History**		
History of any cardiovascular disease	X	X*
Cardiovascular risk factors	X	X*
Family history of cardiovascular disease	X	X
Medication use	X	X*
Stroke related surgical procedures	X	X*
Epilepsy	X	X*
**Neuropsychologic examination**		
Global cognitive function		
Mini Mental State Examination (MMSE)	X	X
Verbal memory function		
Rey Auditory Verbal Learning Test		X
Visuospatial memory		
Rey's Complex Figure Test		X
Speed of information processing		
Symbol-Digit Substitution Task		X
Stroop test		X
Working memory		
Paper and Pencil Memory Scanning Tasks		X
Executive functioning		
Animal Fluency task		X
Attention		
Verbal series attention test		X
Subjective cognitive failures		
Cognitive failures questionnaire		X
Depressive symptoms		
Structured questionnaire depressive symptoms		X
Mini International Neuropsychiatric Interview (MINI)		X
Center of Epidemiological Studies Depression Scale(CES-)		X
Hospital Anxiety and Depression Scale		X
**Physical examination**		
Length and weight	X	X
Waist circumference		X
Blood pressure	X	X
Heart rate	X	X
**Neurological examination**		
Babinsky sign	X	
Sensory system		
Quantitative measurement by vibration tuning fork		X
Muscle strength		
Medical Research Council Scale (MRC)		X
**Mobility and activities of daily living**		
TUG-test		X
Exercise expressed in metabolic equivalent value		X*
Tinetti test (body balance and gait)		X
Modified Ranking Scale (MRS)	X	X
Barthel Index		X
Instrumental activities of daily living questionnaire (IADL)		X
**Additional questionnaires**		
Fatigue		
CIS20R		X
Health related quality of life		
Short Form 36		X
EQ-5D		X
Stroke impact scale 3.0		X
Sleep disturbances		X*
List of Threatening Experiences (LTE)		X*
Work		X*
**Radiological examination**		
Confirmation of index event (CT or MRI)	X	
Angiography	X	
MRI		
T1 magnetization-prepared rapid gradient echo		X
FLAIR pulse sequences		X
Transversal T2* weighted gradient echo sequence		X
Diffusion Tensor imaging		X
Resting state imaging		X
Time-of-flight angiography		X
**Ancillary investigation**		
Electrocardiogram	X	X
Ultrasonography of the carotid arteries	X	X

* Variables were collected both for the period before and after the index event.

Current common rating scales for the severity and cause of stroke did not exist at the time when a substantial proportion of our patients experienced their qualifying event. Therefore a rating of both the severity (NIHSS) and cause (TOAST) was done for all cases retrospectively by a validated approach [9].

### Follow-up

Information on the vital status will be available either from hospital data or through coupling of patient records with data from the municipality registry. All patients alive will be approached for the follow-up assessment according to a two-step approach.

First, all patients will be contacted by letter to inform them about the study; subsequently they will be contacted by phone. In case the patient has moved, the municipality register of the last known residence will be contacted to trace the patient. In cases of an invalid phone number, a second letter will be sent asking the patient to contact our centre to provide a correct phone number. Subsequently, when a patient does not respond to the second letter, the last known general practitioner will be contacted to provide us with updated contact details. The patient will be considered lost to follow-up when known alive, but when untraceable via the procedure described above.

Subsequently, patients will be given the opportunity to participate in an extensive sub study. If they agree to do so, they will be invited to visit our research centre for additional investigations including a structured interview, cognitive assessment, physical and neurological examination, an extensive MRI protocol, an electrocardiogram and an ultrasonography of the carotid arteries (Table 2). In addition, blood samples (serum/plasma/DNA) will be taken for future analysis. When patients are not able to visit our research centre the same investigations will be performed at their homes, except for the ultrasonography of the carotid arteries, electrocardiogram and MRI scan. Controls will undergo the same protocol as patients.

The follow-up has started at the end of 2009 and is planned to finish at the end of 2011.

All these participants signed an informed consent.

### Outcome events

The primary outcome of the study will be all-cause mortality and the composite endpoint of death from all vascular causes; non-fatal stroke, non-fatal (silent) myocardial infarction, cardiovascular procedures (coronary artery bypass grafting, percutaneous transluminal coronary angioplasty, carotid endarterectomy and other arterial revascularization procedures), whichever occurred first. We will perform separate analysis for the occurrence of fatal or non-fatal stroke. Causes of death will be categorized into ischemic stroke, intracerebral hemorrhage, cardiac causes, other vascular causes or non-vascular causes. If we cannot obtain information about the cause of death, the event will be classified as unspecified.

Secondary outcomes are seizures (classified according to the ILAE [10]) and dementia (according to DSM-IV).

Whenever an outcome event is suspected with the aid of a standardized, structured questionnaire, information retrieved will be verified and adjudicated by physicians from the appropriate specialty. In case a patient has died, this information will be retrieved from their general practitioner or a relative. If there is no information available, the event will be classified as a possible event.

### Assessment of variables during follow-up

#### Demographics and life style

Standardized questionnaires on demographics, education (classified using seven categories; one being less than primary school and seven reflecting an academic degree) [11], marital status, living conditions, and life style habits (alcohol consumption, smoking, exercise) will be administered. Alcohol consumption will be defined as units per day and the age at which alcohol consumption had started (and ended if stopped) will be noted. Cigarette smoking behavior will be defined as never, former and current. Subsequently, former and current smoking behavior will be quantified as the number of pack-years, calculated as the number of packs of cigarettes smoked per day multiplied by the number of years a participant had smoked. Exercise will be expressed in the metabolic equivalent value (MET) according to accepted standards [12].

#### Medical history

Structured, standardized questionnaires will be used to assess participants history of hypertension, diabetes mellitus, atrial fibrillation, TIA, stroke, myocardial infarction, coronary artery bypass grafting, percutaneous transluminal coronary angioplasty, carotid endarterectomy and other arterial revascularization procedures [13-16], migraine with or without aura [17], pregnancy and malignancy. Whenever a primary or secondary outcome event is suspected with the aid of this standardized, structured questionnaire, information retrieved will be verified and adjudicated by physicians from the appropriate specialty (see outcome events). The presence of a family history of myocardial infarction, cerebrovascular disease and diabetes mellitus in next of kin will be recorded.

#### Epilepsy

Each patient will be evaluated for a history of epilepsy by means of a standardized, structured questionnaire. Whenever epilepsy is suspected, information will be retrieved from the treating physician and verified and adjudicated by a neurologist (FEdL). Epilepsy will be classified according to the ILAE criteria [10]. Post-stroke epilepsy will be subdivided into early (≤ 7 days post-stroke) and late (> 7 days) post-stroke epilepsy.

#### Current medication

Current medication use and the age at which medication use started will be noted and classified according to the Anatomical Therapeutic Chemical (ATC) classification system. (World Health Organization, WHO Collaborating Centre for drug statistics and methodology, http://www.whocc.no/atcddd/)

#### Neuropsychological assessment

We will administer an extensive neuropsychological test battery that encompasses items from other large-scale epidemiological studies covering the main cognitive domains [18,19]. Global cognitive function will be assessed using the Mini Mental State Examination (MMSE) [20]. Verbal episodic memory function will be assessed by the three-trial version of the Rey Auditory Verbal Learning Test (RAVLT) that also includes a delayed free-recall and recognition trial, a test used to evaluate the ability to acquire and retain new verbal information [7]. Visuospatial episodic memory will be administered by the Rey Complex Figure Test (RCFT), that consists of three trials: a copy trial, an immediate recall trial after 3 minutes and a delayed-recall trial after 30 minutes [21]. To evaluate speed of information processing and executive function, two tests will be used; the abbreviated Stroop Color Word Test (three subtasks, the interference trial measuring response inhibition) [22] and the Symbol-Digit Substitution Task, which is a modified version of the Symbol Digit Modalities Test [23]. A verbal fluency task in which as many animals as possible have to be named within 60 seconds will be used to test semantic memory and executive functioning (response generation). To assess working memory, the Paper and Pencil Memory Scanning Task (four subtasks) [24] will be used. To evaluate attention, the verbal series attention test (VSAT) will be used [25]. To register subjective cognitive failures we will administer the modified Cognitive Failures Questionnaire (CFQ) [26]. The assessments will be carried out under standard circumstances in quiet rooms.

A standardized structured questionnaire used in previous large-scale epidemiological studies will be used to assess the history of depressive symptoms; normal reactions to stressful events or normal grief will carefully be excluded [27]. In case of a depressive episode, age of onset, the medical advice and medication use will be registered. We defined 'depression' as those depressive episodes that have required attention of a general practitioner, psychologist, or psychiatrist. This definition includes minor depression, as well as more severe depression syndromes such as major depression and bipolar depression [27].

In addition participants will be screened for current depressive symptoms by means of the Mini International Neuropsychiatric Interview (MINI), part A, which is a short diagnostic structured interview based on the DSM IV [28]. Additionally, presence of actual depressive symptoms will be assessed by two self report questionnaires, the Centre of Epidemiologic Studies Depression Scale (CES-D) [29] and the Hospital Anxiety and Depression Scale (HADS) [30].

#### Physical and Neurological Examination

Height and weight will be measured without shoes in light clothing. The body mass index (BMI) will be calculated as weight divided by height (in meters) squared. The maximal waist circumference will be measured without shirt, in standing position, between the lowest rib and the iliac crest, at the end of normal expiration [31]. Blood pressure and pulse rate will be measured in triplicate in supine position after 5 minutes rest. Subsequently one measurement is performed after 1 minute in upright position [15].

The strength of the biceps, hand grip, iliopsoas, quadriceps and foot extensor muscles on both sides will be scored according to the medical research council scale (MRC).

The sensory system will be assessed by a quantitative measurement by vibration tuning fork (Rydel-Seiffer^®^) on both first toes and both medial malleolus, also registering ankle edema and the ankle jerk reflex.

#### Gait and balance

We will use a widely used modified version of the original Tinetti test with 17 items: 9 for body balance (score 0-16) and 8 for gait (score 0-12), with a maximum score of 28 [32]. It grades balance while sitting, standing with eyes open and closed, nudging and turning, gait initiation, stride length and width and symmetry. Functional mobility will be classified by using the widely-used TUG-test which is a timed test during which the participant is asked to rise from a standard armchair, walk 3 m, turn, walk back and sit down again [33]. Each participant will perform the test three times.

#### Functional performance

As a measure of disability the Barthel Index and modified Ranking Scale will be used [34]. The activities of daily living will be assessed by the instrumental activities of daily living questionnaire [35].

#### Additional Self-report questionnaires

Several primary sleep disorders are addressed using a number of screening questions. The presence of possible sleep disordered breathing is based on a history of snoring, witnessed sleep-related apneas and non-restorative sleep. Non-REM and REM parasomnias are addressed based on a history of sleepwalking, dream-enacting behavior. Excessive daytime sleepiness will be assessed based on the presence of continuous feelings of sleepiness, sleep attacks or a combination of both. Finally, the presence of sleep-onset and/or sleep-maintenance insomnia is recorded.

For the assessment of fatigue we will use the Checklist on Individual Strength (CIS20R) [36]. The overall health status (quality of life) will be assessed with the Short Form 36 (SF-36) [37,38], the EQ-5D [39] and the Stroke Impact Scale 3 [40].

Adverse life events will be assessed with the 12-item List of Threatening Events (LTE), 6 months before the index event and subsequently the period after the index event [41].

Patients will be asked for their employment status in the month before their index event, within the first year after their index event and at time of the follow-up visit. Each period includes a description of occupation, working hours a week, adjustments in tasks, use of supporting devices and reasons for not working.

#### Ancillary Investigations

##### MRI protocol

MRI scanning will be performed on a 1.5-Tesla Magnetom scanner (Siemens, Erlangen, Germany). The scanning protocol includes whole brain 3D T1 magnetization-prepared rapid gradient-echo (MPRAGE) sequence (TR/TE/TI 2730/2.95/1000 ms; flip angle 7°; voxel size 1.0 × 1.0 × 1.0 mm); FLAIR pulse sequences (TR/TE/TI 12220/85/2200 ms; voxel size 1.0 × 1.2 × 3.0 mm; slice gap 0.6 mm); transversal T2-weighted turbo spin echo sequence (TR/TE 7440/96 ms; voxel size 0.9 × 0.9 × 3.0 mm; slice gap 0.6 mm); Multi-slab 3D time of flight angiography sequence (TR/TE 24/7 ms; voxel size 0.8 × 0.5 × 1.0 mm) will be made of the carotid arteries and the circle of Willis. Gradient echo susceptibility weighted imaging sequence (TR/TE 49/40 ms; voxel size 0.8 × 0.7 × 1.0 mm); DTI (TR/TE 9100/98 ms; voxel size 2.2 × 2.2 × 2.2 mm; 7 unweighted scans, 61 diffusion weighted scans, with non co-linear orientation of the diffusion-weighting gradient, and b value 1000 s/mm^2^) and resting state imaging using a gradient echo EPI (TR/TE 1870/35 ms; voxel size 3.5 × 3.5 × 3.0 mm; slice gap 0.5 mm). During resting state, participants will be told not to concentrate on any particular subject, but just to relax with their eyes closed. The complete scanning protocol takes approximately 60 minutes.

##### ECG

An electrocardiogram (ECG) will be performed and evaluated by a standardized assessment by an experienced cardiologist, registering frequency, cardiac rhythm, cardiac ectopias, cardiac axis, conduction time over the PQ, QRS and QTC intervals, conduction disturbances, left ventricle hypertrophy, pathologic Qs, infarction, repolarisation disturbances and acute ischemia. A final diagnosis is defined as normal, abnormal without clinical significance, abnormal with clinical consequences or pathologic ECG with immediate consultation of a cardiologist when necessary.

##### Carotid ultrasound

A carotid ultrasound assessment will be performed at which the intima media thickness (IMT) will be measured in the distal (near the bulbus) left and right common carotid artery. All measurements will be performed using a phased array real-time scanner (Philips i-u22, The Netherlands) with a 17-5 MHz broadband linear transducer. The IMT will be automatically measured by QLab^® ^qualification software (V. 4.2.1.) according to previously described procedures [42]. All ultrasound measurements will be performed by three experienced and specific trained clinical neurophysiology technicians.

##### Vena puncture

Fasting blood samples will be taken. Immediate analysis will include glucose, creatinine, lipid profile and complete blood count. Additional serum, plasma and DNA will be stored (-80°C) for future biochemical and genetic analyses.

### Statistical analysis

Cumulative risk of primary and secondary outcomes will be estimated with Kaplan-Meier analysis. In the analysis of vascular events, patients who had died from other than the defined fatal endpoints will be censored at the time of death. Cox proportional hazard models will be used to calculate the risk of suffering from any of the primary or secondary outcomes in the follow-up period, with adjustments for the necessary covariates. The relative risk (hazards ratios) will be calculated with their corresponding 95% confidence intervals.

Cross-sectional analysis (for example in the comparison between patients and controls of data acquired during the follow-up) of continuous variables will be done with Student's t test or analysis of variance or in case of skewed distributions which cannot be normalized corresponding nonparametric tests will be used. Chi-squared test will be used for cross-sectional analysis of categorical variables.

## Discussion

Detailed information on risk factors and the long-term prognosis in young stroke patients, and more specific the risk of mortality and recurrent vascular events, remains scarce. These data are often derived from selected patients (often with the exclusion of TIA and hemorrhagic stroke patients) in small sized studies with short follow-up without in person assessment of risk factors and outcomes. We therefore performed the FUTURE study, designed to investigate risk factors and to prospectively assess prognosis in a large cohort of young stroke patients.

Strong elements of our study are the inclusion of both TIA and hemorrhagic and ischemic stroke patients, the very long follow-up (up to 30 years), its sample size of over 1000 potential participants and the availability of baseline data of all consecutive patients in a single university medical centre. In addition, the extensive investigation during a follow-up visit, including advanced neuroimaging has the potential of major contributions to the field. Our study differs from many other young stroke studies due to the inclusion of controls that enable us to compare the frequency of some presumed, but also unknown, risk factors between patients and controls. Detailed risk factor analysis can be done, not only for commonly documented risk factors but also for those that are rarely documented in medical records, like physical inactivity and sleep disturbances. Moreover, the inclusion of healthy controls provides the opportunity to distinguish consequences of a young stroke from other factors like aging effects.

We feel that completion of our study may contribute to a better understanding of the etiology of young stroke and may provide better information for treating physicians and patients with respect to the prognosis of young stroke.

## Competing interests

The authors declare that they have no competing interests.

## Authors' contributions

LRJ, NM, RA, LD, FEDL, EVD: contribution to conception and design; acquisition of data; involvement in drafting the manuscript; final approval of the version to be published

MVA, PS: contribution to conception and design; acquisition of data; revising the manuscript critically; final approval of the version to be published

HS, SO, GD, MVDV, MJ, WVH, RK, MZ, DN: contribution to conception and design; revising the manuscript critically; final approval of the version to be published

## Pre-publication history

The pre-publication history for this paper can be accessed here:

http://www.biomedcentral.com/1471-2377/11/109/prepub
